# The optimal time of starting adjuvant chemotherapy after curative surgery in patients with colorectal cancer

**DOI:** 10.1186/s12885-023-10863-w

**Published:** 2023-05-09

**Authors:** Yuchong Yang, Yao Lu, Hui Tan, Ming Bai, Xia Wang, Shaohua Ge, Tao Ning, Le Zhang, Jingjing Duan, Yansha Sun, Rui Liu, Hongli Li, Yi Ba, Ting Deng

**Affiliations:** 1 Tianjin Medical University Cancer Institute and Hospital, National Clinical Research Center for Cancer, Tianjin’s Clinical Research Center for Cancer, Key Laboratory of Cancer Prevention and Therapy, Huanhuxi Road, Tiyuanbei, Hexi District, Tianjin, 300060 China; 2 Department of Surgical Oncology and General Surgery, The First Affiliated Hospital of China Medical University, Shenyang, Liaoning China; 3 Department of Cancer Center, Peking Union Medical College Hospital, Chinese Academy of Medical Sciences, Beijing, 100730 China

**Keywords:** Adjuvant chemotherapy, Colorectal cancer, Time to chemotherapy, Prognosis

## Abstract

**Background:**

Postoperative adjuvant chemotherapy (AC) is now well-accepted as standard for high-risk stage II and stage III colorectal cancer (CRC) patients, however the optimal time to initiate AC remains elusive.

**Methods:**

A comprehensive literature search was performed using the PubMed and Embase databases. The Hazard ratio (HR) with the corresponding 95% confidence interval (CI) was used as an effect measure to evaluate primary endpoints. All analyses were conducted using Stata software version 12.0 with the Random-effects model.

**Results:**

A total of 30 studies were included in our study. Upon comparison on overall survival (OS), we identified that delaying the initiation of AC for > 8 weeks after operation was significantly associated with poor OS (HR: 1.37; 95% CI: 1.27—1.48; *P* < 0.01). The poor prognostic value of AC delay for > 8 weeks was not undermined by subgroup analysis based on region, tumor site, sample size and study quality. No obvious differences were observed in survival between AC within 5–8 weeks and ≤ 4 weeks (HR: 1.03; 95% CI: 0.96 -1.10; *P* = 0.46). Moreover, two studies both highlighted that the survival benefit of AC was still statistically significant when AC was applied 5–6 months after surgery compared with the non-chemotherapy group.

**Conclusions:**

Delaying the initiation of AC for > 8 weeks after surgery was significantly associated with poor OS. AC started within 8 weeks after surgery brought more benefits to CRC patients. There were no obvious differences in survival benefits between AC within 5–8 weeks and ≤ 4 weeks. Compared to patients not receiving AC after surgery, a delay of approximately 5–6 months was still useful to improve prognosis.

**Supplementary Information:**

The online version contains supplementary material available at 10.1186/s12885-023-10863-w.

## Background

Colorectal cancer (CRC) is one of the most aggressive gastrointestinal tract cancers and the incidence and mortality rank third and second, respectively [1]. For patients with high-risk stage II and stage III CRC, 5-flurouracil-based adjuvant chemotherapy (AC), which has been verified to decrease the risk of recurrence and cancer associated death via eliminating tumor micro-metastasis [2, 3], was recommended to applied following radical surgery by the National Comprehensive Cancer Network (NCCN).

Recently, the result of the International Duration Evaluation of Adjuvant Therapy (IDEA) study fueled interests in specifying the timing and duration of AC after surgery [4, 5]. With no recommendations provided in NCCN guidelines, AC was generally initiated 6–8 weeks after surgery in most clinical trials. However, delays frequently occurred in daily practice for various reasons [6]. Previously, two meta-analyses demonstrated that postponing the postoperative AC was associated with poor survival in CRC patients. Result from Biagi et al*.* showed that each 4 weeks delay result in a 14% decrease of overall survival (OS) [7]. Similarly, Guetz’s study indicated that delaying the initiation of AC for > 8 weeks after operation significantly decreased OS [8]. However, owing to limited research data and small sample size, no consensus has been reached on the actual time to start AC after surgery. Recently, studies with larger sample size has been conducted to investigate the impact of the timing of AC on prognosis in CRC, however the results were inconsistent [9–12]. Therefore, further analysis was needed to instruct clinical practice.

Here in this study, we performed updated meta-analysis, comparing the survival benefits of different time intervals between surgery and postoperative AC (including < 8 weeks *vs.* > 8 weeks and ≤ 4 weeks *vs.* 5–8 weeks), to explore the optimal time to initiate AC after surgery. In addition, based on the existing data, the time point at which postoperative AC was no longer beneficial to CRC patients was discussed.

## Materials and methods

### Literature search

A comprehensive literature search for relevant published studies was performed using the PubMed, Embase databases until February 1st, 2023. The main search terms were “delay”, “interval”, “timing”, “adjuvant chemotherapy”, “colon cancer” and “colorectal cancer”. The references lists of the above articles were also screened.

### Inclusion and exclusion criteria

The eligible studies were enrolled in this study according to PICOS criteria (population, intervention, comparison, outcomes and study design): (1) Population: patients were definitely diagnosed with colon cancer, rectal cancer or colorectal cancer; (2) Intervention: delayed adjuvant chemotherapy; (3) Comparison: colon cancer or colorectal cancer patients with delayed adjuvant chemotherapy in the experimental group versus those without delay in the control group; (4) Outcomes: OS, relapse-free survival (RFS), disease-free survival (DFS), cancer specific-survival (CSS); (5) Study design: comparative studies based on patients with delayed adjuvant chemotherapy and adjuvant chemotherapy without delay. Studies were excluded following exclusion criteria: (1) duplicated articles based on the same patient population or database (studies with different outcomes would be included); (2) studies whose outcome was not reported or impossible to estimate outcomes from the original data; (3) studies with insufficient information (including abstracts or reports from meeting); (4) meta-analyses and reviews.

### Data extraction and assessment of study quality

Two authors reviewed each eligible study and extracted the data independently, and any disagreements were resolved via discussion. Following information was extracted from eligible studies: population year and country, the first author, study design, sample size and source, age and sex of patients, adjuvant chemotherapy regimens, delayed time of adjuvant chemotherapy and survival benefits. The Newcastle–Ottawa scale (NOS) was used to evaluate the quality of eligible studies [13]. Studies with NOS scores ≥ 6 (median score) were assigned as high-quality study (Table S1).

### Statistical analysis

Hazard ratios (HRs) and their 95% confidence intervals (CIs) were used as effect measures to evaluate the primary endpoints (time-to-event outcomes). The method of Tierney was adopted to estimate the HR and 95% CI for those studies in which the HR cannot be extracted directly [14]. All analyses were conducted using the Random-effects model with the method of DerSimonian and Laird. Cochran’s Q test and I^2^ statistics were applied to evaluate statistical heterogeneity. Begg’s and Egger’s tests were used to calculate the effect of publication bias. Sensitivity analysis was performed to assess robustness and reliability of the combined outcomes. All analyses were conducted using the Review Manager 5.2 software (Copenhagen—The Nordic Cochrane Centre; The Cochrane Collaboration, 2012) and STATA software (version 12.0; Stata Corporation, College Station, TX, USA). All statistical tests were two-sided and a P-value of less than 0.05 was considered statistically significant.

## Result

### Search results and study characteristics

**Fig. 1:**
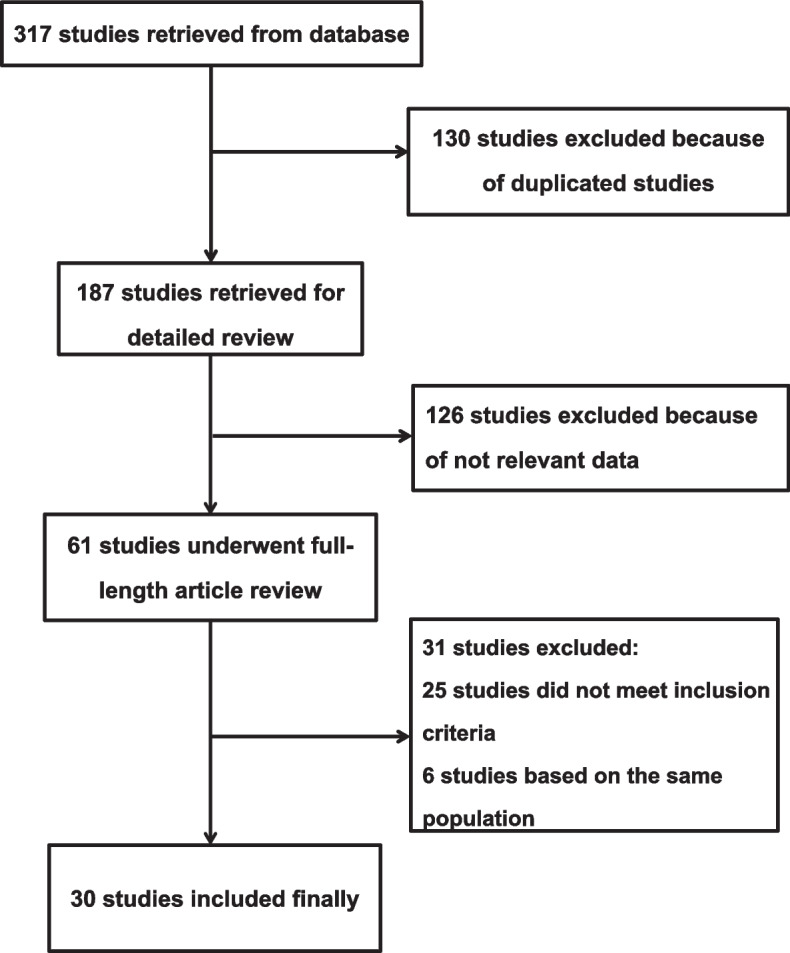
Flow diagram of study selection procedure

A total of 317 potentially relevant articles were initially identified from database searches. Ruling out 130 duplicated studies, 187 studies underwent detailed review. After reviewing the titles and abstracts, 126 studies were further removed. Then 31 studies were excluded based on the inclusion and exclusion criteria after full text review. Finally, a total of 30 studies were included in our study [9–12, 15–40] (Fig. 1).

These studies were published between 2005 and 2023. Aside from seven studies based on Asian populations, 23 studies were performed on Western population (including US, UK, France, Canada, Denmark and Brazil). 29 studies were retrospective cohort studies, and one study was a secondary analysis on a randomized trial. The median sample size was 1053 (Range 102–51,250). Study quality was judged based on the Newcastle–Ottawa Scale (Table S1). The characteristics of these selected studies were summarized in Table 1.

**Table 1:** Baseline characteristics of included studies

Author, Year	Data set	N(M/F)	Site, Stage	Chemotherapy	Endpoint	SQ^a^
Chau, 2005 [15]	Multicenter, UK	801(431/370)	CRC, stage II/III	5FU/LV, 5FU (continuous)	OS	4
Andre, 2007 [16]	Multicenter, France	905 (489/416)	Colon, stage II/III	LV/5FU2, FU + LV	OS,DFS	4
Berglund, 2008 [17]	Norway and Denmark	231(125/106)	Colon, stage III	/	OS	5
Owens, 2009 [19]	MCR, US	3006(1363/1643)	Colon, stage II/III	/	OS	6
Cheung, 2009 [18]	SEER-Medicare, US	6059(3147/2912)	Rectum, stage II/III	/	OS,DFS	5
Ahmed, 2010 [20]	Multicenter,Canada	663 (387/276)	Colon, III, Rectal II, III	5FU based	OS,DFS	7
Bayraktar, 2011 [21]	Multicenter,US	186(72/114)	Colon, stage II/III	5FU based	OS,RFS	8
Czaykowski, 2011 [22]	Multicenter,Canada	345(181/164)	Colon, stage III	5FU/LV	OS,RFS	7
Lima, 2011 [23]	ACR,Canada	1053 (545/508)	Colon, stage III	5FU based	OS	5
Kang, 2013 [24]	Korea	159 (73/86)	CRC, stage III	5FU based	OS,RFS	6
Tevis, 2013 [25]	UWHC, US	355(206/149)	Rectum, stage I-IV	/	OS, LR	7
Yu, 2013 [26]	US	102(45/57)	Colon, stage III	FOLFOX/Xeloda	RFS	5
Day, 2013 [27]	UK	209(118/91)	CRC, Duke A-C	/	OS	7
Tsai, 2013 [40]	Taiwan	1054 (528/526)	CRC, stage III	5-FU-based	OS, CSS	6
Xu, 2014 [28]	SEER-Medicare, US	4209(1954/2225)	Colon, stage II	/	OS, CSS	5
Bos, 2015 [29]	NCR, Netherlands	6620(3530/3090)	Colon, stage III	/	OS	6
Jeong, 2015 [30]	Korea	424(246/178)	Colon, stage II/III	FOLFOX4/FL	DFS	8
Jeong, 2015 [31]	Korea	133(62/71)	Colon, stage III	5FU based	OS,DFS	7
Kim, 2015 [32]	Korea	750(475/275)	CRC, stage II/III	5FU based	OS,RFS	6
Klein, 2015 [33]	DCCG, Denmark	1827(952/875)	Colon, stage III	NR	OS	4
Nachiappan, 2015 [34]	HES, UK	18,306(9889/8417)	Colon, NR	NR	OS	7
Peixoto, 2015 [35]	BCCA, Canada	635(329/306)	Colon, stage III	Oxa-based	RFS, CSS	7
Santos, 2016 [12]	Multicenter, Brazil	1306(643/663)	CRC, stage II/III	NR	OS,DFS	7
Sun, 2016 [9]	NCDB, US	7794(3722/4072)	Colon, stage II/III	NR	OS	6
Kim, 2017 [36]	Multicenter, Korea	5535(3187/2348)	Colon, stage II/III	5FU/Oxa-based	OS	7
Becerra, 2017 [39]	NYSCR, US	1133 (498/635)	Colon, stage III	NR	CSS, OS	7
Gao, 2018 [10]	SEER-Medicare, US	9722(NR)	Colon, stage III	5FU based	OS	4
Turner, 2018 [11]	NCDB, US	51,250 (25,275/25975)	Colon, stage III	NR	OS	5
Choi, 2022 [37]	HIRA, Korea	45,592 (27,148/18444)	CRC, stage II/III	5FU based	OS	6
Farzaneh, 2023 [38]	NCDB, US	8722 (5012/3710)	Rectum, stage II/III	NR	OS	6

*Abbreviations*: *ACR* Alberta Cancer Registry, *BCCA* British Columbia Cancer Agency, *CCS* Cancer specific-survival, *CRC* Colorectal cancer, *DCCG* Danish Colorectal Cancer Group, *F* Female, *DFS* Disease-free survival, *HES* Hospital Episode Statistics, *HR* Hazard ratio, *M* Male, *MCR* Massachusetts Cancer Registry, *N* Number of patients, *NCDB* National Cancer Data Base, *NCR* Netherlands Cancer Registry, *HIRA* Health Insurance Review and Assessment Service Database, *NYSCR* New York State Cancer Registry, *NR* Not report, *OS* Overall survival, *RFS* Relapse-free survival, *SEER* Surveillance, Epidemiology, and End Results, *SQ* Score of study quality, *UWHC* University of Wisconsin Hospitals and Clinics

^a^ Study quality was judged based on the Newcastle–Ottawa Scale

### Quantitative synthesis

####  < 8 weeks vs. > 8 weeks

**Fig. 2:**
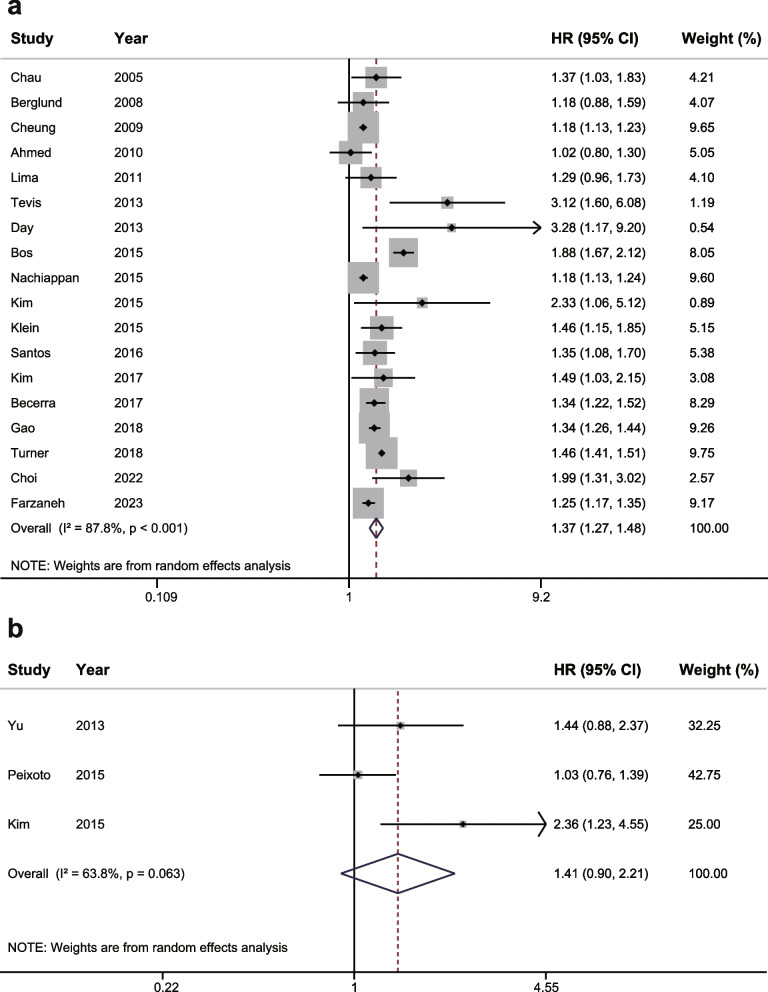
**a** Forest plot assessing overall survival and sharing a common cutoff delay of 8 weeks between surgery and adjuvant chemotherapy. Hazard Ratio (HR) > 1 indicates a worse survival for delayed adjuvant chemotherapy. **b** Forest plot assessing relapse-free survival and sharing a common cut-off delay of 8 weeks between surgery and adjuvant chemotherapy. Hazard Ratio (HR) > 1 indicates a worse survival for delayed adjuvant chemotherapy

For survival comparison, a total of 18 studies can be stratified according to a common cut-off of 8 weeks’ interval between operation and adjuvant chemotherapy. Upon summarizing above studies, we found that the 5-year OS rate was higher in patients receiving AC in < 8 weeks after surgery than those starting the AC in > 8 weeks after surgery (Table S2). Moreover, the result of meta-analysis suggested that postponing the initiation of AC for > 8 weeks after operation was associated with significantly shorter OS (HR: 1.37; 95% CI: 1.27—1.48; *P* < 0.01, Fig. 2A, Table 2). Then the Begg’s and Egger’s tests confirmed the absence of publication bias (Figure S1A, Figure S1B). Sensitivity analysis was performed to evaluate the robustness of our findings. The pooled HR for OS was stable, indicating that the results were reliable (Figure S1C).

**Table 2:** Results of overall and subgroup analyses for effects of initiation of adjuvant chemotherapy for > 8 weeks on survival in patients with colorectal cancer

Categories	N	Patients	Pooled HR (95% CI)	Z value	*P* value	Heterogeneity	
						**I**^**2**^** (%)**	**Ph**
**Overall survival**	18	160,134	1.37 (1.27, 1.48)	7.97	< 0.001	87.80	< 0.001
**Region**							
Asian countries	3	51,877	1.75 (1.35, 2.27)	4.22	< 0.001	0.00	0.448
Non-Asian countries	15	108,257	1.35 (1.24, 1.46)	7.31	< 0.001	89.50	< 0.001
**Tumor site**							
Colon	9	95,677	1.39 (1.26, 1.54)	6.45	< 0.001	90.30	< 0.001
Rectum	3	15,136	1.25 (1.11, 1.41)	3.62	< 0.001	79.40	0.008
**Sample size**							
< 1000	6	3009	1.53 (1.12, 2.09)	2.67	0.008	69.00	0.006
≥ 1000	12	157,125	1.37 (1.26, 1.49)	7.52	< 0.001	91.10	< 0.001
**Study quality**							
< 6	7	70,943	1.32 (1.19, 1.48)	5.12	< 0.001	90.00	< 0.001
≥ 6	11	89,191	1.45 (1.26, 1.65)	5.42	< 0.001	86.40	< 0.001

*Abbreviations*: *CI* Confidence interval, *HR* Hazard ratio, *N* Number of studies, *Ph p* value of Q test for heterogeneity test

**Fig. 3:**
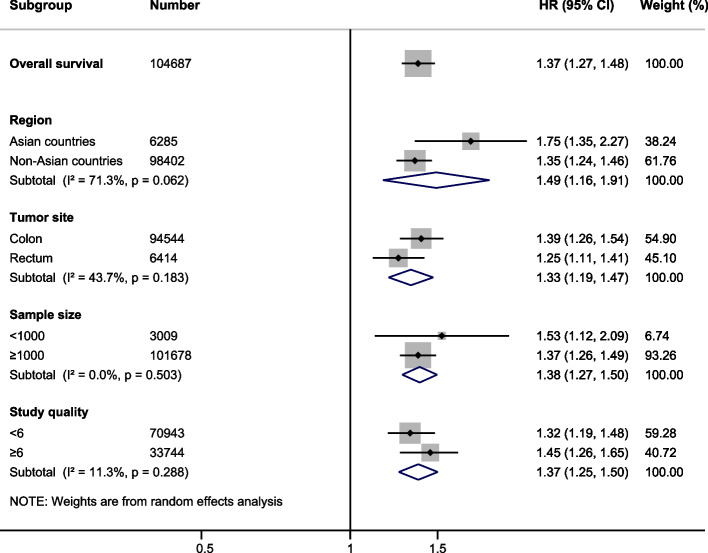
Forest plot of hazard ratios in subgroup analyses by region, tumor site, sample size and study quality comparing overall survival in patients receiving adjuvant chemotherapy in > 8 weeks versus those receiving adjuvant chemotherapy in < 8 weeks after surgery. Hazard Ratio (HR) > 1 indicates a worse survival for delayed adjuvant chemotherapy

Subgroup analyses were stratified by region (Asian vs. non-Asian), tumor site (colon vs. rectum), sample size (≥ 1000 vs. < 1000), and study quality (≥ 6 vs. < 6). Patients who started the AC in < 8 weeks after surgery showed longer OS compared to those receiving the AC in > 8 weeks after surgery in all prespecified clinical subgroups. The pooled results confirmed the poor prognostic role of receiving AC in > 8 weeks after surgery on OS in CRC (Table 2, Fig. 3).

For recurrence investigation, 3 studies can be stratified according to a cut-off of 8 weeks’ interval. The pooled result demonstrated that patients receiving AC in < 8 weeks after surgery had a lower risk of recurrence with longer RFS than those receiving AC in > 8 weeks after surgery, although the difference was not statistically significant (HR: 1.41; 95% CI: 0.90—2.21; *P* = 0.13, Fig. 2B). The results about 5-year RFS of these 3 studies are listed in Table S3.

#### 5–8 weeks vs. ≤ 4 weeks

**Fig. 4:**
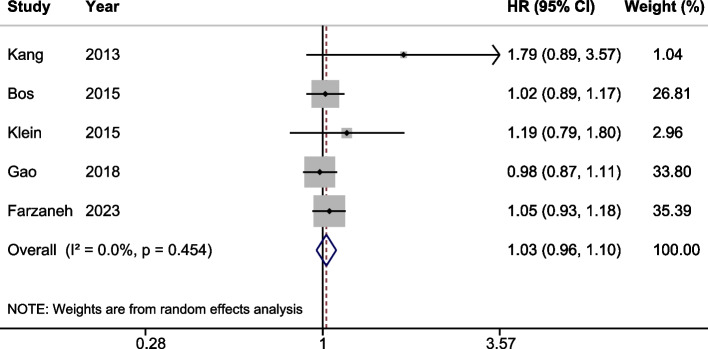
Forest plot assessing comparison of overall survival between starting adjuvant chemotherapy in ≤ 4 weeks and starting adjuvant chemotherapy in 5–8 weeks after surgery. Hazard Ratio (HR) > 1 indicates a worse survival for starting adjuvant chemotherapy in 5–8 weeks after surgery

5 studies were enrolled in the comparison of prognosis following two groups: patients receiving AC in ≤ 4 weeks after surgery and patients starting AC in 5–8 weeks after surgery. We found that compared with receiving AC in ≤ 4 weeks after surgery, starting AC in 5–8 weeks was not significantly associated with OS benefits (HR: 1.03; 95% CI: 0.96 -1.10; *P* = 0.46, Fig. 4). Indeed, the summary table showed that 5-year OS was similar in two groups (Table S4).

## Discussion

According to this updated meta-analysis, delaying initiation of AC after surgery for > 8 weeks was significantly associated with poorer OS. The negative prognostic indication value of delaying initiation of AC for > 8 weeks was not undermined by subgroup analysis based on region, tumor site, sample size and study quality. In addition, publication bias absence verified by Begg’s and Egger’s tests together with sensitivity analysis confirmed the stability and reliability of results from this study.

AC is nowadays well acknowledged as inhibiting tumor growth and prolonging survival after surgery in CRC patients. Some studies reported that trauma by surgery could promote residual tumor growth and tumor metastasis by releasing growth-stimulating factors and triggering immunosuppression [41, 42]. Studies also have shown that surgical trauma can cause an increase in transforming growth factor α (TGFα), which plays an important role in colorectal tumor invasion and metastasis [43, 44]. In vivo experiments on mouse model confirmed that growth factors in healing wounds may promote tumor growth, affecting therapeutic effect of immunotherapy with interleukin-2 (IL-2) and lympokine activated killer (LAK) cells [45, 46]. Therefore, a prolonged interval between operation and AC may lead to the tumor growth and micro-metastases [9]. Moreover, in the blood of some CRC patients after surgery, circulating tumor cells (CTC) or circulating tumor DNA (ctDNA) were detected, and RFS of these patients was significantly reduced [47–50]. About 25% of minimal residual disease (MRD) positive patients can achieve ctDNA clearance through adjuvant infusional fluorouracil, leucovorin, and oxaliplatin chemotherapy, making contributions to improve survival [51, 52].

Important gap remains in our knowledge on mechanical association between the timing of AC and prognosis. There are several possible reasons. Firstly, as mentioned above, residual tumor-promoting cytokines and MRD after surgery are possible drivers for tumor recurrence and metastasis. Given that growth rate is higher in early stage, early intervention with AC may suppress the tumor growth and dissemination [53]. Secondly, with classic mathematical model, Goldie et al*.* demonstrated that aside from mutation rate of tumor cells and sizes, time is also one major contributor to mutation associated drug resistance [54]. Third, Farzaneh et al*.* found that patients in delayed AC group had a higher proportion of positive surgical margin, one important factor for poor prognosis in locally advanced CRC [38]. Harless and colleagues reported the effectiveness of AC was inversely proportional to the interval between surgery and AC initiation [55]. Finally, therapy delay is usually induced due to poor nutritional and performance statuses of patients, which may also contribute to poor clinical outcomes. Whether AC delay is a cause or a consequence of poor prognosis for these patients remains unknown.

Combined with previous studies, our study verified the inverse association of prolonged interval between surgery and AC and survival of patients. While in clinical practice, situation is more complex as therapeutic toxicity may be maximized owning to patient’s poor immune and performance status after operation. Early postoperative intervention may lead to severe chemotherapy-related adverse events and even death [56]. Therefore, the survival benefit of AC may be time-dependent [10]. In parallel with results from another meta-analysis by Guetz et al*. *[8], our study also suggested that AC should be started within 8 weeks after surgery. However, little evidence is available on optimal timepoint to start AC after surgery. Our pooled result showed that 5–8 weeks’ interval between AC and surgery did not increase the risk of mortality compared with that less than 4 weeks, and the 5-year OS was similar between two groups. On the other hand, Bos et al*.* [29] compared the survival of two cohorts, one with 2,950 patients receiving AC 5–6 weeks after surgery and the other with 1,562 patients receiving AC 7–8 weeks after operation, and found that the 5-year OS was slightly higher in the former one (76% vs. 73%). Generally, patients with colorectal surgery need a recovery period of at least 2–4 weeks for wound healing, physical recovery and treatment of postoperative complication. In sum, we hypothesized that 5–6 weeks’ interval may be more reasonable as it may be the optimal choice taking these factors into consideration. Deeper investigations are warranted for further validation.

Until now, the postoperative time point at which AC is no longer beneficial to CRC patients is unclear. In the study performed by Biagi and colleagues, they found that patients starting AC > 12 weeks exhibited better survival compared with patients not receiving AC, and posited a prognostic benefit at 4 or 5 months, which was longer than that commonly recommended in clinical practice [7]. Based on a total of 18,491 patients, Gao’s group showed that the survival benefit of AC was still statistically significant when initiated more than 20 weeks after operation compared with the non-chemotherapy group, thus, AC might be still beneficial with a delay of 5 months [10]. While Turner reported that 489 patients who initiated AC > 24 weeks had a significantly better OS than 20,807 patients who omitted chemotherapy [11]. Therefore, the above mentioned results indicated that AC might still be useful even with a delay of 5–6 months. These results should be confirmed by more studies in the future.

Limitations exist in our study. First, as randomized trial design of postponing AC is neither ethical nor feasible, most of the included studies in this study were retrospective cohort studies, and one was a secondary analysis from a randomized trial. Second, owning to the detailed information availability and limitations of the size and number of studies, we could not perform other subgroup analyses (such as stage III vs. stage II with high-risk features). Third, the cut-offs of time interval among selected studies were different. Besides, with great breakthroughs on CRC postoperative chemotherapy in the last decades, the adjuvant therapy regimen has changed. As 5-fluorouracil-based chemotherapy regimen was mostly used in previous studies while two-drug combination chemotherapy regimen, such as oxaliplatin plus capecitabine, is more commonly used currently. Whether different AC regimens make differences on the choice of initiation time point remains to be determined.

## Conclusions

In conclusion, delaying the initiation of AC for > 8 weeks after surgery was significantly associated with poor OS. AC started within 8 weeks after surgery brought more benefits to CRC patients. There were no obvious differences in survival benefits between AC within 5–8 weeks and ≤ 4 weeks. Compared to patients not receiving AC after surgery, a delay of approximately 5–6 months was still useful to improve prognosis.

## Supplementary Information

**Additional file 1: Figure S1.** Test result for publication bias A. Begg’s test (*P* = 0.13); B. Egger’s test (*P* = 0.36); C. Sensitivity analysis of the hazard ratio of overall survival. **Table S1.** The Newcastle-Ottawa scale quality of included studies. **Table S2.** The summary results about 5-year overall survival in patients receiving AC in < 8 weeks and that in patients who started the AC in > 8 weeks. **Table S3.** The summary results about 5-year relapse-free survival in patients receiving AC in < 8 weeks and that in patients who started the AC in > 8 weeks. **Table S4.** The summary results about 5-year overall survival in patients receiving AC in ≤ 4 weeks and that in patients who started the AC in 5-8 weeks.

## Data Availability

The datasets used during the current study are available from the corresponding author on reasonable request.

## Abbreviations

CRC: Colorectal cancer
NCCN: National Comprehensive Cancer Network
AC: Adjuvant chemotherapy
IDEA: International Duration Evaluation of Adjuvant Therapy
OS: Overall survival
RFS: Relapse-free survival
DFS: Disease-free survival
CSS: Cancer specific-survival
NOS: Newcastle-Ottawa scale
HR: Hazard ratios
CI: Confidence interval
