# Supplementary material for: The optimal time of starting adjuvant chemotherapy after curative surgery in patients with colorectal cancer
Source: BMC Cancer. 2023 May 9;23:422. doi: 10.1186/s12885-023-10863-w (PMC10170689; doi:10.1186/s12885-023-10863-w)
Supplement: Supplementary file 1 — Additional file 1: Figure S1. Test result for publication bias A. Begg’s test (P = 0.13); B. Egger’s test (P = 0.36); C. Sensitivity analysis of the hazard ratio of overall survival. Table S1. The Newcastle-Ottawa scale quality of included studies. Table S2. The summary results about 5-year overall survival in patients receiving AC in < 8 weeks and that in patients who started the AC in > 8 weeks. Table S3. The summary results about 5-year relapse-free survival in patients receiving AC in < 8 weeks and that in patients who started the AC in > 8 weeks. Table S4. The summary results about 5-year overall survival in patients receiving AC in ≤ 4 weeks and that in patients who started the AC in 5-8 weeks. [file 12885_2023_10863_MOESM1_ESM.pdf]

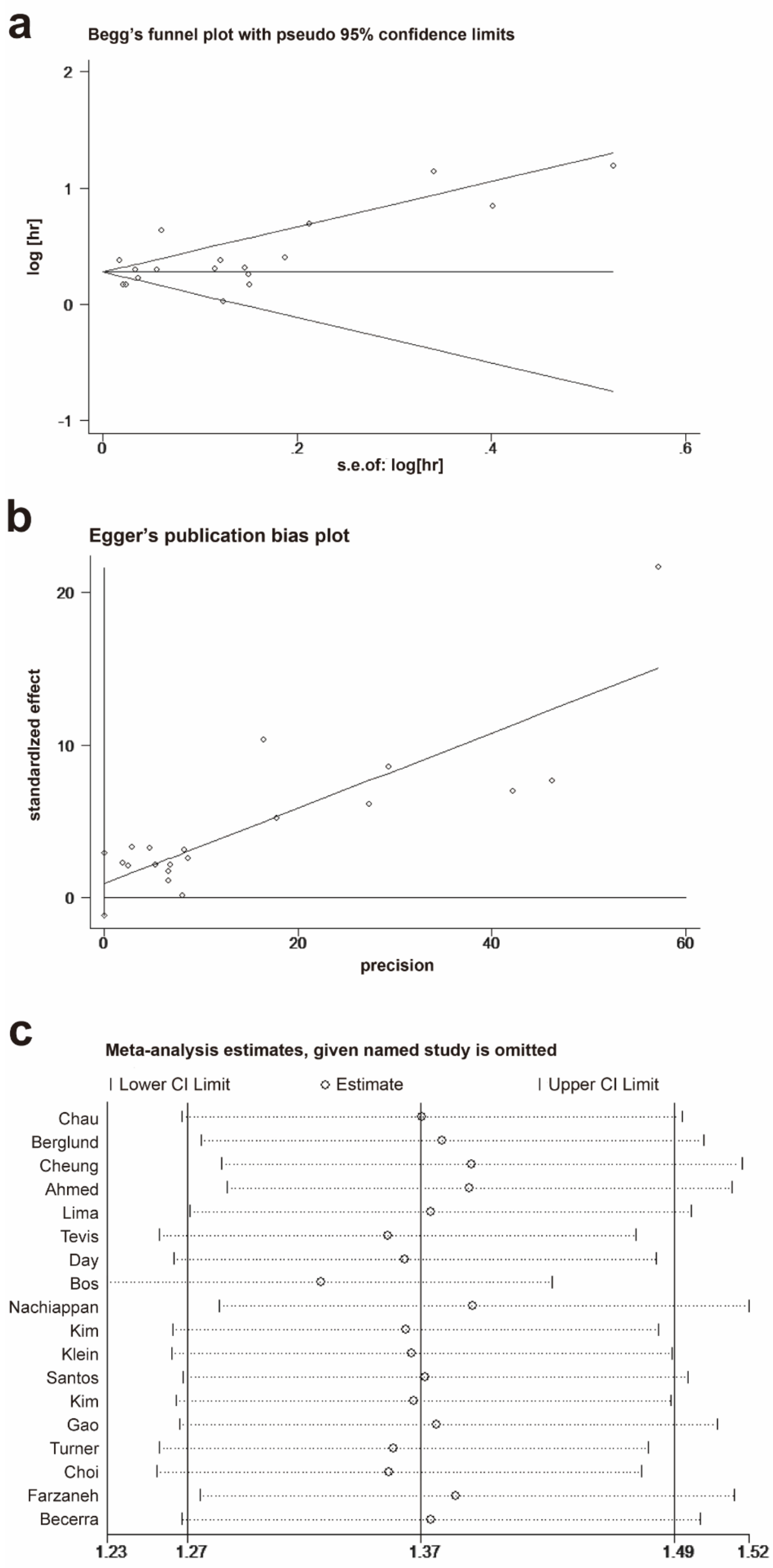

Figure S1. Test result for publication bias A. Begg's test (P = 0.13); B. Egger's test (P = 0.36); C. Sensitivity analysis of the hazard ratio of overall survival.

Table S1. The Newcastle-Ottawa scale quality of included studies.

Study, year	Selection				Comparability		Outcome/Exposure			Total
	REC/ICDA	SNEC/RC	AE/SOC	DO/DC	SC	AF	AO	FU/SMACC	AFU/NRR	
Chau, 2005	0	0	1	1	0	0	1	0	1	4
Andre, 2007	0	0	1	1	0	0	1	1	0	4
Berglund, 2008	0	0	1	1	1	1	0	1	0	5
Owens, 2009	0	1	1	1	1	1	0	1	0	6
Cheung, 2009	0	0	1	1	1	0	1	1	0	5
Ahmed, 2010	1	1	1	1	1	0	0	1	1	7
Bayraktar, 2011	1	1	1	1	1	1	1	1	0	8
Czaykowski, 2011	1	1	1	1	0	1	1	1	0	7
Lima, 2011	1	1	1	1	0	0	0	1	0	5
Kang, 2013	1	1	1	1	0	1	0	1	0	6
Tevis, 2013	1	1	1	1	1	1	0	1	0	7
Yu, 2013	1	1	1	1	0	0	0	1	0	5
Day, 2013	1	1	1	1	1	1	0	1	0	7
Tsai, 2013	1	1	1	1	0	0	1	1	0	6
Xu, 2014	0	1	1	1	0	1	0	1	0	5
Bos, 2015	1	1	1	1	0	1	0	1	0	6
Jeong, 2015	1	1	1	1	1	1	1	1	0	8
Jeong, 2015	1	1	1	1	1	1	0	1	0	7
Kim, 2015	1	1	1	1	1	0	0	1	0	6
Klein, 2015	0	1	1	1	0	0	0	1	0	4
Nachiappan, 2015	1	1	1	1	0	1	0	1	1	7
Peixoto,2015	1	1	1	1	0	1	0	1	1	7
Santos, 2016	1	1	1	1	1	1	0	1	0	7
Sun, 2016	1	1	1	1	0	1	0	1	0	6
Kim, 2017	1	1	1	1	0	1	1	1	0	7
Becerra, 2017	1	1	1	1	0	1	1	1	0	7
Gao, 2018	0	1	1	1	0	0	0	1	0	4
Turner, 2018	1	1	1	1	0	0	0	1	0	5
Choi, 2022	1	1	1	1	0	0	1	1	0	6
Farzaneh, 2023	1	1	1	1	0	0	1	1	0	6

Abbreviations, AE: ascertainment of exposure; AF: study controls for any additional factors; AFU: adequacy of follow-up of cohorts; AO: assessment of outcome; DO: demonstration that outcome of interest was not present at start of study; FU: follow-up long enough (36 months) for outcomes to occur; ICDA: Is the case definition adequate; RC: Representativeness of the cases; REC: representativeness of the exposed cohort; SC: study controls for age, sex; "1" means that the study is satisfied the item and "0" means the opposite situation; SNEC: selection of the non-exposed cohort; SOC: Selection of controls; DC: Definition of controls; SMACC: Same method of ascertainment for cases and control; NRR: Non-Response rate.

Table S2. The summary results about 5-year overall survival in patients receiving AC in < 8 weeks and that in patients who started the AC in > 8 weeks.

Author, Year	Initiation of AC for < 8 weeks		Initiation of AC for > 8 weeks	
	N	5 y-OS	N	5 y-OS
Chau, 2005	/	77%	/	72%
Berglund, 2008	149	57%	82	52%
Ahmed, 2010	422	60%	241	57%
Cheung, 2009	4476	60%	1583	53%
Lima, 2011	286	71%	767	52%
Tevis, 2013	110	86%	55	61%
Day, 2013	102	90%	107	74%
Kim, 2015	566	80%	31	57%
Klein, 2015	1539	75%	288	66%
Bos, 2015	5618	74%	1002	60%
Nachiappan, 2015	1549	69%	1341	64%
Santos, 2016	606	74%	712	68%
Becerra, 2017	730	68%	403	61%
Gao, 2018	6911	62%	2811	53%
Turner, 2018	38117	73%	13133	64%
Choi, 2022	22655	83%	1367	70%
Farzaneh, 2023	5526	73%	3196	66%

Abbreviations, 5y-OS: 5 years overall survival; AC: adjuvant chemotherapy; N: number of patients.

Table S3. The summary results about 5-year relapse-free survival in patients receiving AC in < 8 weeks and that in patients who started the AC in > 8 weeks.

Author,Year	Initiation of AC for < 8 weeks		Initiation of AC for > 8 weeks	
	N	5 y-RFS	N	5 y-RFS
Yu, 2013	58	66%	44	57%
Kim, 2015	566	71%	31	40%
Peixoto,2015	291	71%	344	72%

Abbreviations, 5y-RFS: 5 years relapse-free survival; AC: adjuvant chemotherapy; N: number of patients.

Table S4. The summary results about 5-year overall survival in patients receiving AC in ≤ 4 weeks and that in patients who started the AC in 5-8 weeks.

Author,Year	Initiation of AC for ≤ 4 weeks		Initiation of AC for 5-8 weeks	
	N	5 y-OS	N	5 y-OS
Kang, 2013	126	71%	33	55%
Bos, 2015	1106	75%	4512	75%
Klein, 2015	219	83%	1320	80%
Gao, 2018	746	62%	6165	62%
Farzaneh, 2023	905	71%	4621	73%

Abbreviations, 5y-OS: 5 years overall survival; AC: adjuvant chemotherapy; N: number of patients.
